# Retraction: Paricio, A.; Lopez-Carmona, M.A. MuTraff: A Smart-City Multi-Map Traffic Routing Framework. *Sensors* 2019, *19*, 5342

**DOI:** 10.3390/s20133759

**Published:** 2020-07-04

**Authors:** Sensors Editorial Office

**Affiliations:** MDPI, St. Alban-Anlage 66, 4052 Basel, Switzerland; sensors@mdpi.com

It has been brought to our attention that the methods and results presented in [1] are almost identical to those previously published in [2]. The paper [1] will therefore be marked as retracted.

MDPI is a member of the Committee on Publication Ethics and takes seriously its responsibility to publish only high-quality research. We regret that this was not discovered prior to publication and offer our apologies to the readers of *Sensors*.
